# Synergistic and Antagonistic Effects of Mixed-Leaf Litter Decomposition on Nutrient Cycling

**DOI:** 10.3390/plants13223204

**Published:** 2024-11-15

**Authors:** Vestine Mukamparirwa, Salim M. S. Maliondo, Canisius Patrick Mugunga

**Affiliations:** 1 Regional Research School in Forest Sciences (REFOREST), College of Forestry, Wildlife and Tourism, Sokoine University of Agriculture, Chuo Kikuu, Morogoro P.O. Box 3009, Tanzania; 2 Department of Ecosystems and Conservation, Sokoine University of Agriculture, Morogoro P.O. Box 3010, Tanzania; maliondo@sua.ac.tz; 3 Department of Agricultural Engineering, Rwanda Polytechnic, Huye College, Huye P.O. Box 330, Rwanda; 4 Department of Forestry and Nature Conservation, University of Rwanda, Musanze P.O. Box 210, Rwanda; mugungacp@gmail.com

**Keywords:** mixed-species litter, litter decomposition, synergistic, antagonistic effects, soil fertility

## Abstract

Understanding decomposition patterns of mixed-leaf litter from agroforestry species is crucial, as leaf litter in ecosystems naturally occurs as mixtures rather than as separate individual species. We hypothesized that litter mixtures with larger trait divergence would lead to faster mass loss and more balanced nutrient release compared to single-species litter. Specifically, we expected mixtures containing nutrient-rich species to exhibit synergistic effects, resulting in faster decay rates and sustained nutrient release, while mixtures with nutrient-poor species would demonstrate antagonistic effects, slowing decomposition. We conducted a mesocosm experiment using a custom wooden setup filled with soil, and the litterbag method was used to test various leaf litter mixtures. The study involved leaf litter from six agroforestry tree species: three species from humid highland regions and three from semi-arid regions. Treatments included three single-species leaf litter mixtures, three two-species mixtures, and one three-species mixture, based on the sampling region. Species included *Calliandra calothyrsus* (Ca), *Croton megalocarpus* (Cr), *Grevillea robusta* (G), *Alnus acuminata* (A), *Markhamia lutea* (M), and *Eucalyptus globulus* (E). Decay rate constants (k) were estimated using non-linear least-squares regression and observed mass loss was compared to predicted values for mixed-species litter treatments to assess synergistic and antagonistic effects. A two-way linear mixed-effects model was employed to explain variation in mass loss. Results indicate positive non-additive effects for leaf litter mixtures including nutrient-rich species and negative non-additive effects for mixtures including nutrient-poor species. The mixture of Ca + Cr + G had positive non-additive or synergistic effects as it decomposed faster than its corresponding single-species litter. Leaf litters with higher lignin content, such as A + M + E and Ca + Cr + G, exhibited less lignin release compared to what would be expected based on individual litter types, demonstrating antagonistic effects. These findings highlight that both litter nutrient constituents and litter diversity play an important role in decomposition processes and therefore in the restoration of the degraded and nutrient-depleted soils of Rwanda.

## 1. Introduction

Litter from trees, crops, and other plants is typically mixed and decomposed together in the soil [1]. However, the quality and quantity of residues produced by each plant species differ and, in turn, affect the rate of decomposition and nutrient release [2]. The recent literature suggests that decomposition processes are influenced by a combination of physical, chemical, and biological factors, especially when leaf litter from various species is mixed [3]. This interspecific mixing alters the chemical content and modifies the physical characteristics of the litter surface, thereby affecting decomposition dynamics [4]. Studies highlight that such alterations can significantly influence the abundance and activity of decomposers. Litter mixtures from different species create unique microenvironments that can either facilitate or hinder microbial colonization and activity, depending on the specific combinations of chemical compounds present [5].

The rate of litter decomposition and nutrient release are affected by the species variety in the litter mixture. Agroforestry species exhibit diverse litter qualities, which are distinguished by their specific carbon-to-nitrogen ratios, lignin concentrations, and other chemical features. These differences impact the activity of microorganisms and the speed at which decomposition occurs, ultimately affecting the way nutrients are processed in the soil [6].

In an agroecosystem, the decomposition and nutrient release patterns from litter can exhibit non-additive effects. This means the actual outcome in mixed litter differs from the predicted outcome based on individual species in monoculture. Non-additive effects can be synergistic, where the observed effect is greater than expected, or antagonistic, where the observed effect is less than expected [7,8]. Nevertheless, the non-additive effects in litter mixture decomposition are not yet fully understood [9,10]. The nutrient transfer hypothesis often explains non-additive mass loss in litter mixtures and suggests that decomposers favor high-nitrogen (N) litters, releasing N that can then be transferred to low-N litter. This process accelerates the decomposition of more resistant litter, leading to non-additive mass loss by increasing the overall decomposition rate [11]. According to [12], the soil fauna can accelerate carbon (C) and nitrogen (N) release by improving litter quality across different elevations, thereby enhancing the decomposition process. On the other hand, a study conducted in *Eucalyptus* and *Acacia* plantations found that changes in microbial community structure and diversity did not affect decomposition rates, suggesting that nutrient transfer between litter types might not always lead to non-additive effects in decomposition [13].

In Rwanda, a recent study showed that the plantation of exotic species, particularly *Eucalyptus* species, negatively affects the soil while native trees improve soil properties and microbial processes [14]. The study revealed that there was increased N mineralization under *Eucalyptus maidenii*, despite reports on the detrimental effects of *Eucalyptus* species on the growth and activity of soil microorganisms, due to their soil-acidifying effects and secretion of allelopathic compounds [14].

According to [15], in Rwanda, *Eucalyptus* species dominate forest plantations, accounting for 89%. Pines make up 6.5%, mixed exotic forests constitute 3.1%, and plantations of native species represent only 1.4%. From these concepts, we hypothesized that studying these species and considering their decomposition dynamics from a mixed-litter perspective could provide more insight into how litter species mixtures can promote nutrient release in an agroforestry ecosystem during the decomposition process.

Despite the evident benefits of litter mixtures for nutrient cycling, this area has received limited research attention in eastern Africa and in Rwanda in particular. There is a lack of comprehensive studies examining the decomposition dynamics of mixed litter from different agroforestry species and this study aims to address this gap by determining the effects of mixed-leaf litter of selected tree species on decomposition and nutrient release patterns in Rwanda.

In our study, we explored the potential of enhancing decomposition rates of diverse quality leaf litter, including a few native and exotic species that are already adopted in agroforestry landscapes, to improve organic resource management. By focusing on mixed-species litter decomposition, we aim to provide insights for better agroforestry practices and address the existing research gap. Most decomposition studies on mixed litter reported mass losses and nitrogen dynamics [16]. Far less attention has been given to the release of other nutrients during decomposition processes and no studies, so far, reported on litter decomposition dynamics based on mixtures of agroforestry tree species in Rwanda. This study compared litter decomposition rates of native tree species (*Markhamia lutea*, *Croton megalocarpus*), exotic tree species (*Eucalyptus globulus*, *Grevillea robusta*), and exotic N_2_-fixing tree species (*Alnus acuminata*, *Calliandra calothyrsus*). We tested the following hypotheses:

1. A larger trait divergence of the litter quality of the species in a mixture results in a faster mass loss of the mixture than expected based on the single species;
2. Mixed leaf litter results in a more balanced and sustained nutrient release compared to single-species leaf biomass, due to complementary decomposition rates and nutrient profiles.

## 2. Results

### 2.1. Observed and Predicted Decay Rate Constants

After 120 days of the experimental setup, the combined mass loss was found to be affected by the mixture of leaf litter. The observed and predicted decay rate constants (*k*) and the mass remaining for single-species and mixed litters are shown in Table 1. The table shows a comparison between the observed and predicted *k* and the percentage of mass remaining (% M.R.) for all treatments as well as their respective coefficients of determination (R²), which indicate the goodness of fit of the model. The observed decay rates (*k*) and the percentage of mass remaining were measured directly from the experimental data, reflecting the actual decomposition processes occurring under the studied conditions while the predicted values were derived using a single exponential decay model that assumes a constant decay rate over time. The observed decay rates are influenced by the optimum moisture content and quality of the litter.

The comparison reveals how well the model predictions align with the observed data. Treatments involving combinations of three species, such as Ca + Cr + G and A + M + E, generally show higher decay rates and lower mass remaining, reflecting faster decomposition. The results showed that observed *k* values were higher than predicted across all litter mixtures, and we observed a high *k* rate in both treatments with three-species mixtures compared to when a species is decomposing alone.

**Figure 1:**
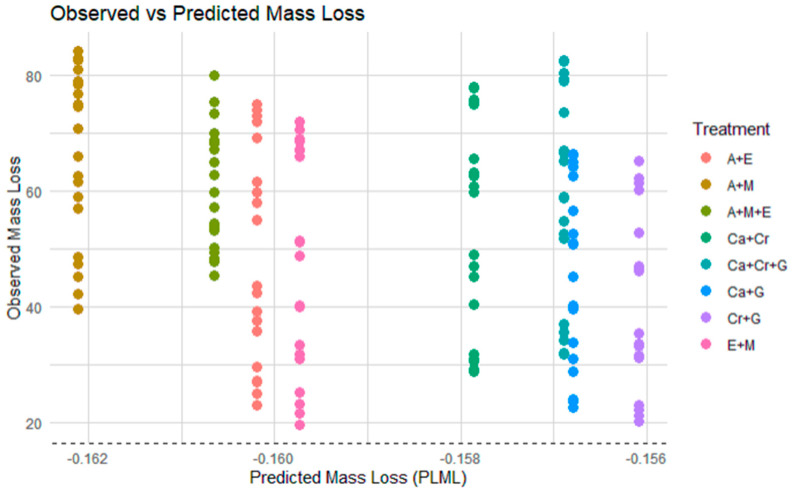
Comparison of observed vs. predicted mass loss for different litter mixtures: The plot compares the observed mass loss (%) to the predicted mass loss (PLML) (%) for study litter mixtures across treatments. The dashed line at the bottom represents the theoretical line of equality, indicating no points align, reinforcing the presence of enhanced decomposition rates in mixed-species litterbags.

The exponential decay model was fitted to perform the interaction effect analysis by plotting observed mass loss with predicted mass loss. Figure 1 shows that the line of equality (where observed mass loss equals predicted mass loss) is positioned at the bottom of the plot, below all of the data points. This indicates that all observed mass loss values are significantly higher than the predicted mass loss values. It implies a strong synergistic effect in all mixtures. The actual decomposition rates are faster than what would be predicted if species decomposed independently without any interactions. The results from the exponential decay rate show that when species are mixed, they enhance each other’s decomposition rates more than expected.

### 2.2. Mass Lossover Time 

**Figure 2:**
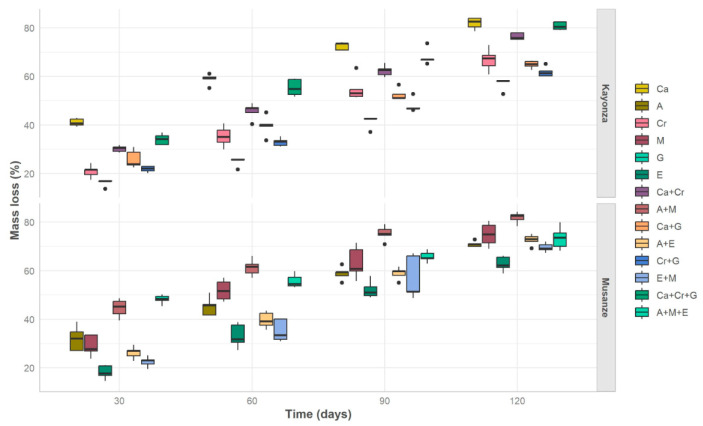
Boxplots (Q1, median, Q3) with the observed measures on mass loss based on tree species basing on their respective sampled areas and time taken to decompose.

**Figure 3:**
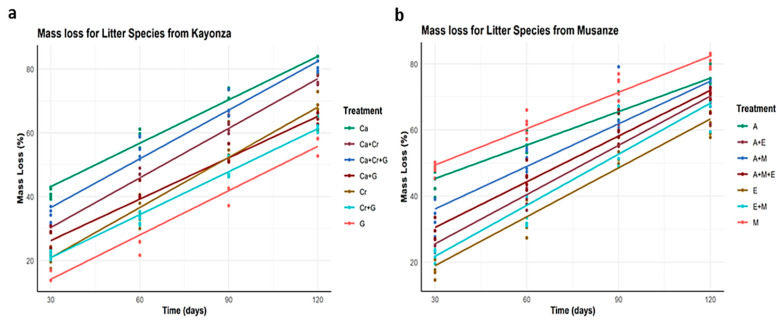
The mass loss model predicted estimates based on the interaction of input variables (tree species and time taken to decompose) based on the two sites where the litter is collected from, (**a**) Kayonza and (**b**) Musanze. The data were log transformed.

**Figure 4:**
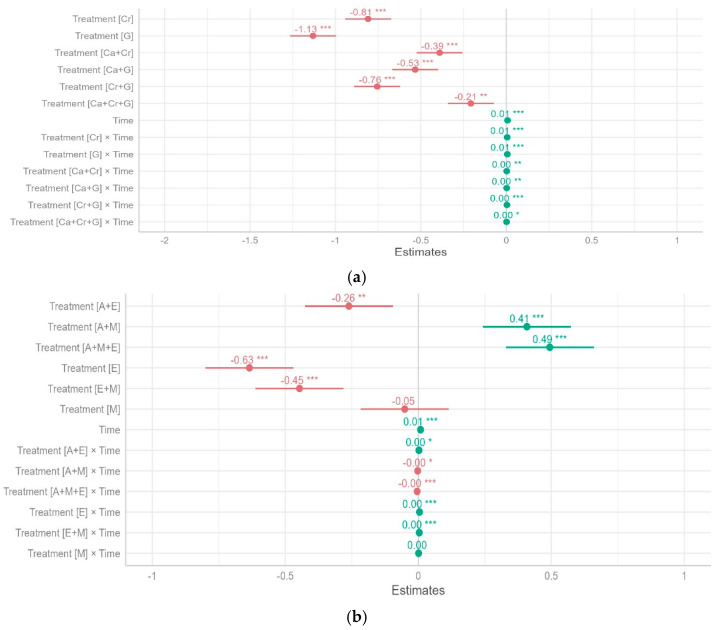
Fixed effects standardized estimates of the two-way linear mixed-effects model explaining variation in mass loss of selected agroforestry tree species from the sampling areas of (**a**) Kayonza and (**b**) Musanze and their associated 95% confidence intervals. Asterisks indicate the significance level of the corresponding *p*-values (*** *p* < 0.001, ** *p* < 0.01, * *p* < 0.05). The effects Treatment and Time are in relation to their baseline levels, *C. calothyrsus* (**a**) and *A. acuminata* (**b**).

Our mixed model predictions for repeated measures showed that the main effects of litter species identity and time (Figure 2 and Figure 3) significantly affected litter mass loss (Figure 4a,b). There were significant differences in single-species litter mass loss among the six species compared to their mixtures (Figure 3a,b). Across the four time intervals and among the treatments with tree litter collected from Kayonza, *C. calothyrsus* had the greatest litter mass loss, followed by the mixture of all three litters (Ca + Cr + G), and *G. robusta* had the lowest litter mass loss (Figure 3a). The model indicates that *C. calothyrsus*, as a baseline species (Figure 4a), exhibits a significantly higher decomposition rate compared to other species. This rapid decomposition is statistically significant (*** *p* < 0.001), underscoring the influence of its nitrogen-fixing trait, which enhances microbial activity and accelerates litter breakdown. The strong synergistic effects observed in the mixtures (Ca + Cr; Ca + G) are further validated by significant positive estimates, highlighting the facilitative role of *C. calothyrsus* in promoting decomposition in mixed-species treatments.

For the treatments with tree litter collected from Musanze, the native species *M. lutea* decomposed most, followed by *A. acuminata*, the mixture of the two litters (A + M), and the three-litter mixture (A + M + E). *E globulus* had the lowest litter mass loss (Figure 3b). The model indicates that the litter mixtures which include *M. lutea* and *A. acuminata* (A + M) and (A + M + E) exhibit a significantly higher decomposition rate compared to other species (Figure 4b).

The model results showed that all mixture treatments with more proportions of slow-decomposing litter species showed lower mass losses compared to the ones having fewer proportions. The mixtures *C. megalocarpus* + *G. robusta* (Cr + G), *C. calothyrsus* + *G. robusta* (Ca + G), *E. globulus + M. lutea* (E + M), and *A. acuminata* + *E. globulus* (A + E) had 41.13%, 45.74%, 46.06%, and 49.37% mass loss, respectively (Figure 3a,b, Table 2).

For the mixing effect on litter mass loss, the decomposition rate constants of both *E. globulus* and *G. robusta* litter in the mixed litterbags increased compared with the pure litterbags (Table 2). This effect is highlighted (Figure 3a,b) in three-mixture treatments, where Ca + Cr + G was the second treatment with a high decomposition rate compared to others in Kayonza, while A + E + M, A + E, and E + M decomposed faster compared to pure E treatments in Musanze.

For Kayonza, the results showed that there are significant non-additive mixing effects on litter mass loss with all mixture species and their interactions with the time taken to decompose. The effect of leaf litter on mass loss varied between −1.13 (*G. robusta*) to −0.21 (Ca + Cr + G), indicating that litter mixtures decomposed slower than expected as they showed negative litter mixing effects on the litter decomposition rate (Figure 3a).

Both *C. megalocarpus* and *G. robus*ta show significant differences in decomposition rates compared to the baseline (*C. callothyrsus*). The model results reveal significant negative estimates for these species (* *p* < 0.05) (Figure 3a), suggesting slower decomposition rates, likely due to their higher lignin content (Figure 6E). However, the significant interaction terms for mixtures involving *C. megalocarpus* and *G. robus*ta (e.g., Cr + G, *** *p* < 0.001) indicate that while individually slower decomposers, these species contribute to enhanced decomposition dynamics when combined with other litter types (Figure 3a).

For Musanze, the effect of leaf litter on mass loss varied between −0.63 (*E. globulus*) to −0.05 (*M. lutea*), indicating that species litter decomposed more slowly than anticipated, exhibiting negative effects of litter mixing on the decomposition rate (Table A1). A positive effect was observed for A + M and A + M + E with estimates of 0.41 and 0.49, respectively. All of the interactions were close to zero, with values between 0.00 and 0.01 (Table A1), indicating that the effect of time intervals considered for the leaf litter to decompose in either single-species litters or mixtures was not significantly different to what was expected.

### 2.3. Nutrient Release

**Figure 5:**
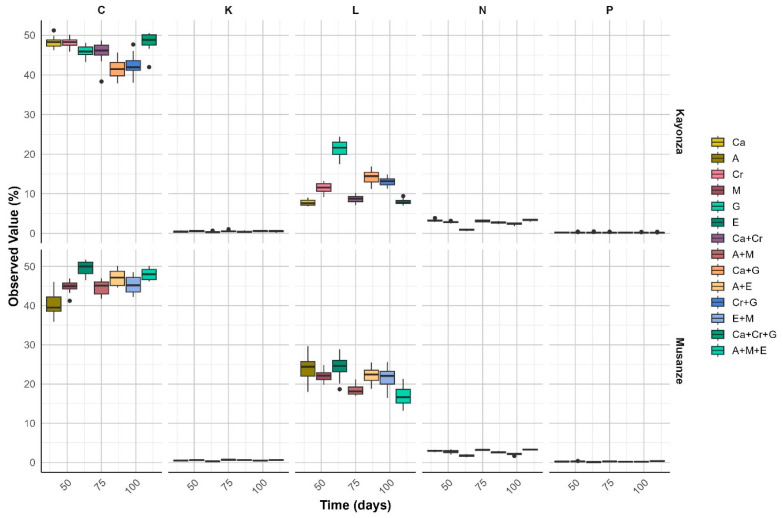
Observed nutrient release across the study. N, P, K, C, and lignin were the measured nutrients to predict the quality of selected species.

**Figure 6:**
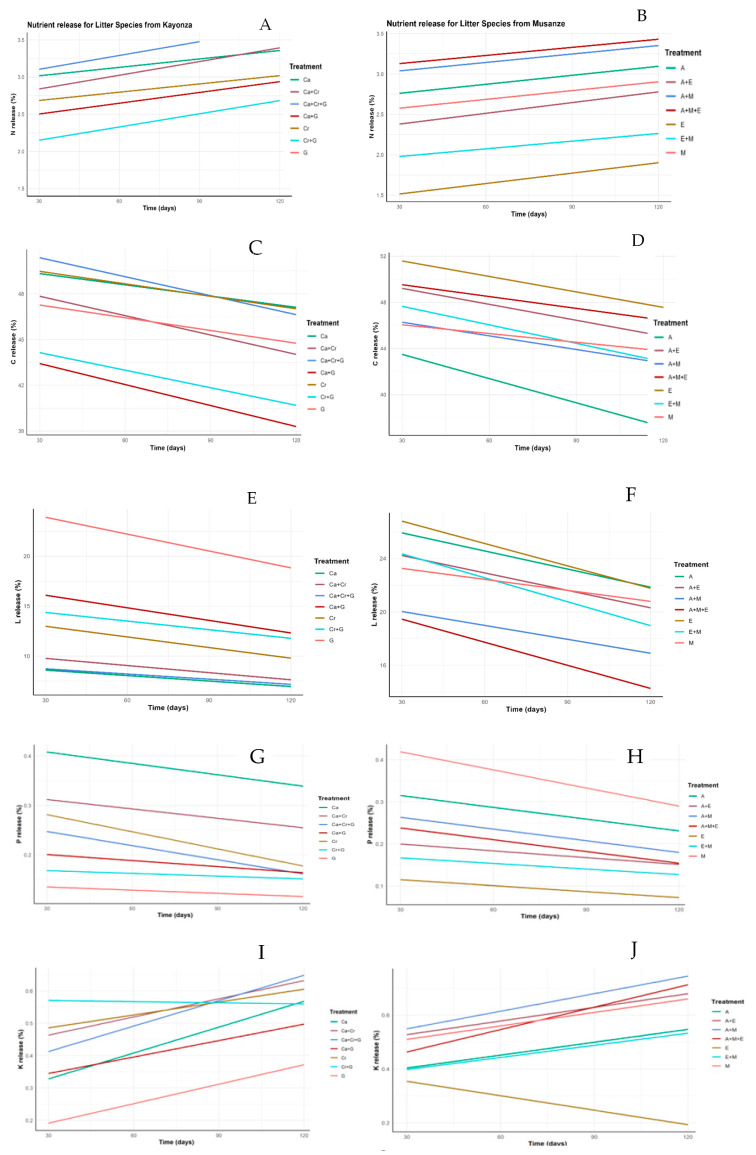
Plots showing the change in nutrient release (%) (**A**–**J**) on the predicted estimates based on the interaction of input variables (single tree species or mixtures and time) across the treatments of litters collected from the Kayonza and Musanze sites. The released nutrients are nitrogen (N), phosphorus (P), potassium (K), carbon (C), and lignin (L.) after a 120-day interval of incubation.

There were variations in the percentage of nutrients released across the 120 days of decomposition, as shown in Figure 5. The potassium (K) and phosphorus (P) released were minimal and showed little variation across treatments and time, implying that these nutrients were immobilized. In our experiment, the observed nutrients released differed from those predicted for the mixed litterbags (Figure 5 and Figure 6), implying that the litter of different species does not decompose independently. As for our second hypothesis, we found that mixing the leaf litter of two to three species increased the decomposition constant of the nutrient-poor soil due to complementary decomposition rates and nutrient profiles.

The N release remained consistently low for *G. robusta* and *E. globulus* as the slow-decomposing species compared to when they are mixed with other species. The carbon concentration in the remaining litter decreased slightly as decomposition proceeded. This was observed for the remaining litter of *A. acuminata*, *M. lutea,* and their mixture with equal ratios.

## 3. Discussion

### 3.1. Mass Loss

Previous studies found that litter mixing usually generates synergistic non-additive effects more frequently than antagonistic non-additive effects on litter decomposition [17]. Our study also found that litter mixing promoted the decomposition of more than 80% of the mixture (Ca + Cr + G) at 120 days after incubation. The mass loss of two slow-decomposing litter species, *G. robusta* and *E. globulus,* was accelerated when mixed with native and N_2_-fixing-species litter. This supported our first hypothesis that the larger trait divergence of the litter quality of the species in a mixture results in a faster litter mass loss of the mixture than expected based on the single species. Many studies report non-additive mass loss in litter mixtures [18]. Our results categorically showed that decay rates *k*, calculated on an individual litter basis or combined basis (Table 1), were enhanced when mixed. Translocation of limiting nutrients, e.g., nitrogen, from nutrient-rich litter to nutrient-poor litter, i.e., *C. calothyrsus* to *G. robusta,* could influence the decay rate. It is hypothesized that the presence of another litter component with different physical attributes may improve the microclimate of the decaying environment through better aeration while permitting nutrient translocation [2]. The study of [19] revealed that mixing the leaf litter of the two species increased the decomposition constant of the nutrient-poor litter (*V. paradoxa*); the decomposition constant of the nutrient rich litter (*F. albida*) also increased in the mixed litterbags.

*E. globulus* is characterized by slower decomposition rates, indicating a lower decomposition rate relative to *A. acuminata*. This slower rate is likely due to its high lignin and polyphenol content, which inhibits microbial activity [20]. However, significant positive interaction effects in mixtures with *M. lutea* (E + M) suggest that while *E. globulus* is slow to decompose alone, its combination with other species can mitigate these effects, enhancing overall decomposition rates. This result is similar to [20], who reported that the mixture of *E. globulus* and *Acacia mearnsii* accelerated decomposition and the nitrogen cycle, and the species interactions were most obvious under a 50:50 mixture.

### 3.2. Nutrients

The model explaining nutrient release showed the following results across different leaf litter species, and treatment combinations revealing notable patterns in both species sampled from Kayonza and Musanze. In the treatments of the species from the Kayonza region, carbon (C) release is consistently high across all treatments, indicating robust decomposition activity, especially in the three-litter-species combination (C + Cr + G). This suggests that multi-species mixtures could be highly effective in enhancing soil organic matter in semi-arid regions.

In contrast, potassium (K) and phosphorus (P) releases are minimal and show little variation across treatments and time, implying that these nutrients are either being immobilized or are less affected by the decomposition processes within the treatments. This finding is consistent with previous studies that have shown limited potassium and phosphorus availability in the early stages of litter breakdown, especially when initial nutrient content is low [21,22].

The release of lignin is significantly higher in Musanze, particularly in mixtures involving native species like *M. lutea* and the N_2_-fixing *A. acuminata* (A + M). This could be attributed to higher microbial activity in the humid highlands, as well as the presence of two species that are highly effective in promoting decomposition. These results support the hypothesis that litter mixtures enhance the breakdown of recalcitrant compounds, such as lignin, by creating a more favorable environment for microbial activity [23]. The contrasting chemical and physical properties of the mixed species likely stimulate diverse microbial communities, accelerating the decomposition of complex compounds [24]. However, the release of N remains consistently low across all treatments and sites, suggesting that N is tightly bound within the plant tissues. This could be explained by the immobilization of N within the highly structured organic compounds formed during humification. As litter decomposes, organic matter is transformed into humic substances, which are stable and resistant to microbial breakdown [25]. These humic substances can bind N, thereby reducing its availability and delaying its mineralization. This immobilization process is further reinforced by the slow mineralization of P, which can also become incorporated into these stable organic compounds [26].

Additionally, the formation of lignin-derived compounds during humification further immobilizes these nutrients, inhibiting their release into the soil. This complex interaction between litter quality, nutrient availability, and microbial activity highlights the role of humification in stabilizing nutrients in mixed-litter systems [27]. The immobilization of N and slow P mineralization observed in this study reflect the creation of these structured organic compounds, which play a significant role in regulating nutrient cycling in agroforestry systems [26,27].

Similar previous findings by [27] showed that the reduced quality of individual litter would cause immobilization of nutrients in a mixed composition. The low-quality litter in a species mixture can also promote nutrient retention in an ecosystem, providing nutrient resources for future decay processes and long-term nutrient storage in litter layers [2]. Leaf litters with high N contents (low C-to-N ratios) decomposed more rapidly than leaf litters with low N contents [19]. According to [28], a non-additive effect on N release was found in the poplar-dominant litter mixtures; N release in poplar litter was accelerated in the mixture compared to the monoculture, highlighting that microbial organisms preferentially exploit N nutrient released from higher quality litter, whereas lower quality litter immobilizes N and provides a resource for further decay. In our study, we found it would be easy for the N-rich *M. lutea,* the native species litter, to translocate N to the N-poor *E. globulus*. This partially supported our second hypothesis that enhanced nutrient release in mixed litter is partially supported by increased lignin and carbon dynamics; the slower release of nitrogen and phosphorus suggests that nutrient availability is complex and may depend on factors such as initial litter quality and species composition. These findings indicated that while litter mixtures offer potential benefits, certain nutrients may remain constrained, requiring further exploration of longer-term decomposition dynamics and nutrient cycling.

As implied, mixing various litters increases the complexity of the litter types and nutrient sources and changes the microenvironment, which alters the physical, chemical, and biological functions of the litter decomposition process and ultimately affects litter decomposition [29].

## 4. Materials and Methods

### 4.1. Study Site

**Figure 7:**
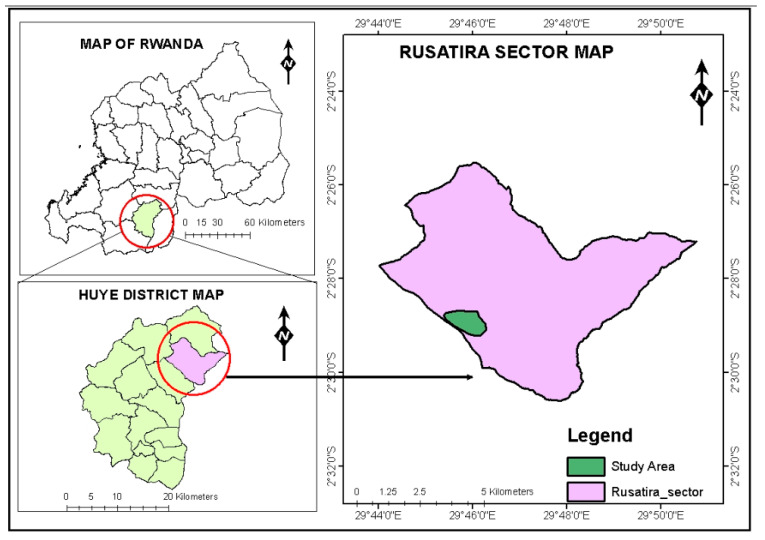
Map of the study area.

This study was conducted at the Rubona agricultural research station in the Huye district, southern province of Rwanda (Figure 7). The station is located at an altitude of 1650 m. a.s.l., latitude S 2°28′59.59″ and longitude E 29°46′22.46″ [30], in the mid-altitude agro-ecological zones [31].

The area has a bimodal rainfall distribution, with a lot of rain from March to June and less rain from October to December. The minimum annual temperature is 14.8 °C, while the maximum temperature is 25.8 °C [32]. The leaves were sampled from two contrasting ecoregions, Musanze (1°29′59.42″ S, 29°38′5.89″ E.) located in the northern humid highlands [33], and Kayonza (1°51′ S, 30°39′ E,) located in the eastern semi-arid region [34].

### 4.2. Experimental Design

The greenhouse/mesocosm was set up in June 2022. Mature green leaves were sampled from the following tree species—*Alnus acuminata*, *Markhamia lutea*, and *Eucalyptus globulus* from Musanze, and *Calliandra calothyrsus*, *Croton megalocarpus,* and *Grevillea robusta* from Kayonza. Thus, five replicates were prepared for each litter or litter mixture. The wooden mesocosm system served as an incubator chamber. The leaves were air-dried and thoroughly mixed. Sub-samples from each litter type were then dried at 60 °C for 48 h to measure moisture content. According to the methods for estimating litter decomposition [35], we used 50 g of litter for each single species and each litter mixture placed in the litterbags. The bag dimension of the litterbags was 30 × 20 cm; they were made using nylon fabric with a mesh size of 1.5 mm. The litter treatments (T) were designed to investigate the effects of single- (100% of one species; that is, 1:0 ratio), two- (50% of each species; that is, 1:1 ratio), and three-species litter mixtures (33.3% of each species; that is, 1:1:1 ratio) on litter mass loss and nutrient release. Specifically, single-species litterbags were used to generate decomposition rates and nutrient release for pure litter. These rates were used as a baseline for comparison with mixed-species litterbags to determine if interaction effects occurred when litters of different species were mixed. Thus, seven treatments with litter species from the same sampling area included T1: *A. acuminata* (A); T2: *M. lutea* (M); T3: *E. globulus* (E); T4: *A. acuminata*/*M. lutea* (A + M); T5: *A. acuminata/E. globulus* (A + E); *T6*: *M. lutea*/*E. globulus* (M + E); and T7: *A. acuminata*/*M. lutea*/*E. globulus* (A + M + E) collected from Musanze. While for litter collected from Kayonza, the treatments were T1: *C. calothyrsus* (C); T2: *C. megalocarpus* (Cr); T3: *G. robusta* (G); T4: *C. calothyrsus*/*C. megalocarpus* (C + Cr); T5: *C. calothyrsus/G. robusta* (C + G); T6: *C. megalocarpus*/*G. robusta* (Cr + G); and T7: *C. calothyrsus*/*C. megalocarpus*/*G. robusta* (C + Cr + G).

**Figure 8:**
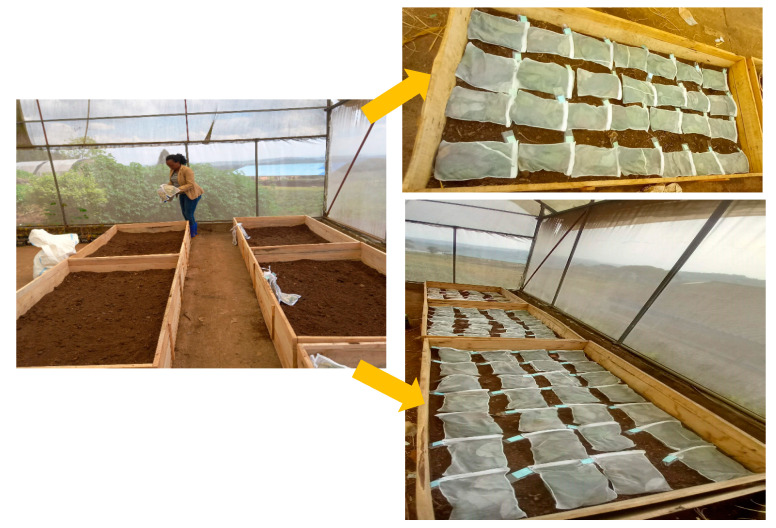
The experimental layout used to study the mass loss and nutrient release from seven treatments made by single-species litters and litter mixtures from six agroforestry species.

A fully randomized block design (n = 5) resulted in 280 bags in total. There was a total of 7 litter treatments (single species and mixtures), 4 retrieval times (30, 60, 90, and 120 days), 2 sample areas (semi-arid and humid highland), and 5 blocks per treatment per sampling time. This design allowed for the collection of 5 litterbags for each treatment at each retrieval time, ensuring a robust sampling size for statistical analysis. When retrieving the bags at each given time, litter was individually sealed in plastic bags and was immediately brought to the laboratory for analysis (Figure 8).

The litterbags were carefully cleaned to remove any remaining soil particles and then dried in an oven at 60 °C for 48 h to stop any further decomposition. The litterbags were then weighed to calculate the relative mass loss for each species, expressed as a percentage of their initial mass. To maintain consistent soil moisture levels and ensure uniform conditions for all litter samples, soil moisture content was maintained in the mesocosm through daily manual watering using a graduated watering can to field capacity with distilled water and regular moisture checks conducted by feeling the soil.

### 4.3. Variables Measured

#### 4.3.1. Mass Loss and Decomposition Rate Constant k

1. The observed mass loss of leaf samples was determined by comparing the initial air-dry mass with the remaining air-dry mass after a specified period. The percentage of both single-species and mixed-species litter mass loss was calculated as below:

Observed litter mass loss % = (M_b_ − M_a_)/M_b_ * 100 (1)

where M_b_ is the litter mass in the litterbags before decomposition and M_a_ is the litter mass remaining after decomposition.

2. To assess the decomposition rate and amounts of nutrients released, the species litter remaining in each litterbag retrieved on each sampling occasion was weighed separately. The percentage dry weight of decomposed leaf litter at time t, Rt (%), was calculated as follows:

Rt (%) = M_t_/M_o_ × 100 (2)

where M_t_ is the dry weight of decomposed leaf litter at time t and M_0_ is the initial dry weight in the litter bag.

3. To describe the decomposition pattern and calculate decomposition rate constants (*k*), data for each species in each treatment were modeled using a single exponential model [36]:

M_t_/M_o_ = exp^(−*k*t)^ (3)

where M_t_ and M_0_ are defined as above in (2), exp is the base of the natural logarithm, *k* is the decay rate coefficient, and *t* is the time of placement in days.

4. To determine whether interactions in litter mixtures occurred, the predicted mass loss for a litter mixture is calculated based on the individual mass loss rates of the species involved, adjusted for their proportional contributions to the mixture. The calculation assumes that there are no interactions between the litter species in the mixture, meaning that each species decomposes independently of the others. This was calculated as follows according to [37], where PLML stands for predicted litter mass loss:

PLML % = ∑ (Wi × Mi)(4)

where Wi is the proportion (or weight fraction) of species i in the litter mixture and Mi is the mass loss percentage of species iii when decomposed individually, usually expressed as a percentage of the original mass.

5. Observed mass loss for the mixture: The actual mass loss observed in the mixed-species litterbag is measured directly from the experiment.
6. Comparison of predicted vs. observed mass loss: The analysis compares the predicted mass loss (PLML) to the observed mass loss of the litter mixture to determine if there is a difference.

When the observed mass loss equals the predicted litter mass loss (PLML), it suggests that species in the mixture decompose independently, with no interactions. If the observed mass loss exceeds the PLML, it indicates a positive or synergistic effect, where decomposition is faster than expected. Conversely, if the observed mass loss is lower than the PLML, this points to a negative or antagonistic effect, with decomposition slower than predicted. The strength of the interaction is measured using the formula 1 − (observed remaining mass/predicted remaining mass), as defined by [37].

#### 4.3.2. Chemical Analysis of Litter Samples

The litter chemistry was determined at the Rwanda Agriculture and Animal Resources Development Board laboratory (RAB). The oven-dried leaf samples (at 60 °C) of individual and mixed species were grounded and processed for chemical characterization (N, P, K, C, and lignin). Plant nutrients (N, P, and K) were analyzed through the complete oxidation of a 1 g sample by Kjedahl digestion using sulfuric acid, hydrogen peroxide, and selenium digestion mixture [38]. Nitrogen and potassium were determined from 5 mL aliquot of the digestion mixture using an autoanalyzer (Thermo Fisher Scientific, Waltham, MA, USA).

Phosphorus was determined by adding ammonium molybdate and ascorbic acid and the absorbance read at 420 nm. Total C was determined by oxidation with concentrated sulfuric acid and 1 M aqueous potassium dichromate mixture with external heating [39]. Lignin concentrations were determined following the Klason method after hydrolysis with 72% H_2_SO_4_ [40].

#### 4.3.3. Statistical Analysis

The observed and predicted mass loss of individual litter types and combined mass loss were calculated using the single negative exponential decay model [36]. The decay rate constant *k* of each component litter was classified by species. To fit the exponential decay model, we used the nlsLM() function from the minpack.lm package (version 1.2-4) in R software (R Core Team, 2022). All analyses were performed in the R Studio environment (R Studio version 4.3.2). The nlsLM function was chosen for its robust handling of nonlinear least squares optimization, especially for complex decay models where initial parameter estimates are essential. The predicted values for each mixture were calculated pro rata using the species proportions to obtain 100% litter. Data for mass loss and nutrient release at each sampling occasion were analyzed using a linear mixed-effects model that fits well for repeated measures data in R software [41]. The effects of our treatments (seven litter species, single or mixture) were tested for the interactions between them, and the time taken to decompose (fixed effects) on the litter mass loss and nutrient release. We used the block as a random effect. Before statistical analysis, the data were tested for normality and homogeneity, and transformation was used in the case of no homogeneity. After the general ANOVA hypothesis was verified, detailed post hoc mean comparisons of significant differences in the litter variables among the treatments were performed using Tukey’s HSD. The sampling time and the litter mixture treatments were considered fixed factors to examine the effects of the two factors on the release of nutrients. The predicted values were derived using a single exponential decay model that assumes a constant decay rate over time.

## 5. Conclusions

This study demonstrates the synergistic effects of mixing leaf litter on decomposition, highlighting how mixtures of litter types with contrasting chemical properties enhance decay rates. Specifically, mixtures of *A. acuminata* and *M. lutea* showed the highest mean mass loss at 65.77% ± 3.35, suggesting strong complementary interactions between these species. Similarly, the combination of *A. acuminata*, *M. lutea*, and *E. globulus* achieved a 60.78% ± 2.29 mass loss, further supporting the role of diverse species combinations in accelerating decomposition.

On the other hand, the mixture of *C. calothyrsus* and *C. megalocarpus* resulted in a 53.68% ± 4.02 mass loss, whereas the three-species mixture of *C. calothyrsus*, *C. megalocarpus*, and *G. robusta* resulted in 59.54% ± 3.99, indicating that species with varying substrate qualities can significantly enhance mass loss through complementary interactions. The results align with previous studies, which demonstrated that litters with better initial substrate quality, such as *C. calothyrsus* and *M. lutea*, can positively affect litters with poorer substrate quality, like *G. robusta* and *E. globulus*. These combinations significantly influenced nutrient release, especially in Musanze, where mixtures of the native species *M. lutea* with *A. acuminata* and *E. globulus* showed lower lignin release compared to *E. globulus* alone, which had higher lignin release. This is likely due to complementary interactions that promote greater microbial diversity and activity.

In agroforestry systems, understanding these non-additive litter effects is crucial for selecting optimal plant mixtures. Mixtures of native species, such as *M. lutea* and *A. acuminata*, not only improve nutrient cycling but also contribute to long-term soil fertility. The ability to maximize nutrient release through carefully selected species combinations can support restoration and rehabilitation efforts in degraded areas, contributing to sustainable nutrient cycling and enhanced soil development.

Future research should include long-term monitoring of microbial biomass and biota abundance to provide deeper insights into the mechanisms driving nutrient cycling in mixed-species litter. This understanding will be key to optimizing agroforestry practices, improving carbon sequestration, and reducing greenhouse gas emissions.

## Figures and Tables

**Table 1:** Comparison of the combined observed and predicted decay rates, *k*, and percent mass remaining (%M.R.) for individual and mixed-leaf litters under greenhouse conditions.

Site	Treatment	Observed		M.R. (%)	Predicted		M.R. (%)
		k	R^2^		K	R^2^	
Kayonza	Ca	0.959	0.917	36.42 ± 1.28	0.864	0.867	40.06 ± 0.70
Kayonza	Cr	0.613	0.988	55.51 ± 0.25	0.552	0.938	61.06 ± 1.23
Kayonza	G	0.474	0.913	65.04 ± 1.00	0.427	0.863	71.54 ± 1.10
Kayonza	Ca + Cr	0.775	0.833	46.32 ± 0.94	0.697	0.783	50.96 ± 1.37
Kayonza	Ca + G	0.662	0.851	54.26 ± 0.54	0.596	0.801	59.69 ± 1.43
Kayonza	Cr + G	0.581	0.925	58.87 ± 0.70	0.523	0.875	64.76 ± 0.76
Kayonza	Ca + Cr + G	0.872	0.859	40.46 ± 0.47	0.784	0.809	44.51 ± 0.89
Musanze	A	0.767	0.878	48.24 ± 1.15	0.69	0.828	53.06 ± 0.85
Musanze	M	0.791	0.826	45.2 ± 0.41	0.712	0.776	49.71 ± 0.42
Musanze	E	0.566	0.981	58.41 ± 1.52	0.509	0.931	64.25 ± 1.93
Musanze	A + M	1.007	0.998	34.23 ± 0.55	0.906	0.948	37.65 ± 0.36
Musanze	A + E	0.699	0.997	50.63 ± 0.42	0.629	0.947	55.69 ± 0.38
Musanze	E + M	0.638	0.941	53.94 ± 1.13	0.574	0.891	59.33 ± 0.65
Musanze	A + M + E	1.044	0.857	32.03 ± 1.46	0.939	0.807	35.24 ± 1.01

Coefficients of determination (R^2^) indicate how the data fit the single negative exponential model. Treatments: *C. calothyrsus* (Ca), *C. megalocarpus* (Cr), *G. robusta* (G), *C. calothyrsus* + *C. megalocarpus* (Ca + Cr), *C. calothyrsus* + *G. robusta (*Ca + G), *C. megalocarpus* + *G. robusta* (Cr + G), *and C. calothyrsus* + *C. megalocarpus* + *G. robusta* (Ca + Cr + G) for litter collected from Kayonza. *A. acuminata* (A), *M. lutea* (M), *E. globulus* (E), *A. acuminata* + *M. lutea* (A + M), *A. acuminata* + *E. globulus* (A + E), *M. lutea* + *E. globulus* (M + E), *and A. acuminata* + *M. lutea* + *E. globulus* (A + M + E) collected from Musanze.

**Table 2:** Summary table for mass loss of all mixed-litter treatments.

Treatment	Mean Mass Loss (%)
*A. acuminata* + *E. globulus* (A + E)	49.37 ± 4.11
*A. acuminata* + *M. lutea* (A + M)	65.77 ± 3.35
*E. globulus + M. lutea* (E + M)	46.06 ± 4.33
*A. acuminata* + *M. lutea* + *E. globulus* (A + M + E)	60.78 ± 2.29
*C. calothyrsus* + *G. robusta* (Ca + G)	45.74 ± 3.39
*C. calothyrsus* + *C. megalocarpus* (Ca + Cr)	53.68 ± 4.02
*C. megalocarpus* + *G. robusta* (Cr + G)	41.13 ± 3.49
*C. calothyrsus* + *C. megalocarpus* + *G. robusta* (Ca + Cr + G)	59.54 ± 3.99

## Data Availability

All of the data supporting the reported results can be found in this article.

## Appendix A

**Table A1:** Log-transformed fixed effects (Treatment and Time) standardized estimates of the two-way linear mixed-effects model explaining variation in mass loss of selected agroforestry tree species from the sampling areas of Kayonza and Musanze and their associated 95% confidence intervals. The effects Treatment and Time are in relation to their baseline levels (*C. calothyrsus* and *A. acuminata*).

Litter Species from Kayonza			Litter Species from Musanze		
Predictors	Estimates (CI)	*p*	Predictors	Estimates (CI)	*p*
(Intercept)	3.58 (3.48–3.67)	<0.001	(Intercept)	3.68(3.56–3.79)	<0.001
Treatment [Ca + Cr]	−0.39(−0.52–−0.26)	<0.001	Treatment [A + E]	−0.68−0.84–−0.51	<0.001
Treatment [Ca + Cr + G]	−0.21(−0.34–−0.07)	0.003	Treatment [A + M]	−0.31(−0.47–−0.14)	<0.001
Treatment [Ca + G]	−0.53(−0.67–−0.40)	<0.001	Treatment [A + M + E]	−0.49(−0.66–−0.33)	<0.001
Treatment [Cr]	−0.81(−0.94–−0.67)	<0.001	Treatment [E]	−1.03(−1.20–−0.87)	<0.001
Treatment [Cr + G]	−0.76(−0.89–−0.62)	<0.001	Treatment [E + M]	−0.85(−1.01–−0.68)	<0.001
Treatment [G]	−1.13(−1.27–−1.00)	<0.001	Treatment [M]	0.08(−0.08–0.25)	0.321
Time	0.01(0.01–0.01)	<0.001	Time	0.01(0.00–0.01)	<0.001
Treatment [Ca + Cr] × Time	0.00(0.00–0.00)	0.002	Treatment [A + E] × Time	0.01(0.00–0.01)	<0.001
Treatment [Ca + Cr + G] × Time	0.00(0.00–0.00)	0.043	Treatment [A + M] × Time	0.00 (0.00–0.00)	0.009
Treatment [Ca + G] × Time	0.00(0.00–0.00)	0.003	Treatment [A + M + E] × Time	0.00 (0.00–0.01)	<0.001
Treatment [Cr] × Time	0.01 (0.00–0.01)	<0.001	Treatment [E] × Time	0.01 (0.01– 0.01)	<0.001
Treatment [Cr + G] × Time	0.00 (0.00–0.01)	<0.001	Treatment [E + M] × Time	0.01(0.00–0.01)	<0.001
Treatment [G] × Time	0.01 (0.00–0.01)	<0.001	Treatment [M] × Time	0.00 (−0.00–0.00)	0.978
**Random Effects**					
σ^2^	0.01			σ^2^	0.01
τ_00 Block_	0.00			τ_00 Block_	0.00
ICC	0.00			ICC	-
N _Block_	5			N _Block_	5
Observations	140			Observations	140
Marginal R^2^/Conditional R^2^	0.959/0.959			Marginal R^2^/Conditional R^2^	0.925/NA
